# *N*-(Phenyl­sulfon­yl)naphtho­[2,1-*b*]furan-1-carboxamide

**DOI:** 10.1107/S1600536811050495

**Published:** 2011-11-30

**Authors:** M. Shetprakash, P. A. Suchetan, B. S. Palakshamurthy, K. M. Mahadevan, V. P. Vaidya

**Affiliations:** a Center for Advanced Materials and Department of Chemistry, Tumkur University, Tumkur, Karnataka 572 103, India; b Department of PG Studies and Research in Physics, Tumkur University, Tumkur, Karnataka 572 103, India; c Department of Chemistry, Kuvempu University, Shankaraghatta, Shimoga, Karnataka, India

## Abstract

In the title compound, C_19_H_13_NO_4_S, the mol­ecule is twisted at the S atom with a C—S—N—C torsion angle of −65.2 (2)° between the benzene ring and the –SO_2_—NH—C=O segment. The dihedral angle between the benzene and the naphtho­furan ring system is 83.3 (1)°. In the crystal, mol­ecules are linked by N—H⋯O hydrogen bonds into chains running along the *c* axis. An intra­molecular N—H⋯O(furan) inter­action is also observed.

## Related literature

For related structures, see: Gowda *et al.* (2009 ▶, 2010 ▶).
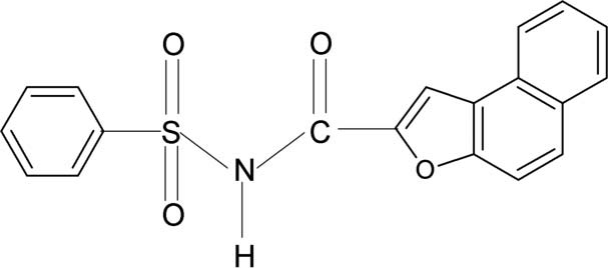


## Experimental

### 

#### Crystal data

C_19_H_13_NO_4_S
*M* *_r_* = 351.36
Monoclinic, 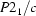

*a* = 13.8504 (10) Å
*b* = 12.2166 (8) Å
*c* = 9.7164 (6) Å
β = 101.248 (2)°
*V* = 1612.48 (19) Å^3^
*Z* = 4
Mo *K*α radiation
μ = 0.23 mm^−1^
*T* = 299 K
0.35 × 0.3 × 0.25 mm

#### Data collection

Bruker APEXII CCD area-detector diffractometer
Absorption correction: multi-scan (*SADABS*; Sheldrick, 1996 ▶) *T*_min_ = 0.924, *T*_max_ = 0.945
15289 measured reflections
2986 independent reflections
2394 reflections with *I* > 2σ(*I*)
*R*_int_ = 0.027

#### Refinement

*R*[*F* ^2^ > 2σ(*F* ^2^)] = 0.041
*wR*(*F* ^2^) = 0.133
*S* = 0.85
2986 reflections
231 parameters
1 restraint
H atoms treated by a mixture of independent and constrained refinement
Δρ_max_ = 0.25 e Å^−3^
Δρ_min_ = −0.38 e Å^−3^

### 

Data collection: *APEX2* (Bruker, 2004 ▶); cell refinement: *APEX2* and *SAINT-Plus* (Bruker, 2004 ▶); data reduction: *SAINT-Plus* and *XPREP* (Bruker, 2004 ▶); program(s) used to solve structure: *SHELXS97* (Sheldrick, 2008 ▶); program(s) used to refine structure: *SHELXL97* (Sheldrick, 2008 ▶); molecular graphics: *ORTEP-3* (Farrugia, 1997 ▶); software used to prepare material for publication: *SHELXL97*.

## Supplementary Material

Crystal structure: contains datablock(s) I, global. DOI: 10.1107/S1600536811050495/ds2146sup1.cif

Structure factors: contains datablock(s) I. DOI: 10.1107/S1600536811050495/ds2146Isup2.hkl

Supplementary material file. DOI: 10.1107/S1600536811050495/ds2146Isup3.cml

Additional supplementary materials:  crystallographic information; 3D view; checkCIF report

## Figures and Tables

**Table 1:** Hydrogen-bond geometry (Å, °)

*D*—H⋯*A*	*D*—H	H⋯*A*	*D*⋯*A*	*D*—H⋯*A*
N1—H1*N*⋯O4	0.79 (3)	2.33 (3)	2.653 (2)	106 (2)
N1—H1*N*⋯O2^i^	0.79 (3)	2.66 (1)	3.057 (2)	113 (2)

Symmetry code: (i) 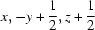.

**Fig. 1.**
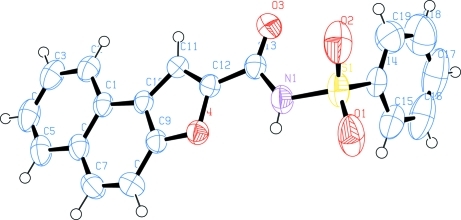
Molecular structure of the title compound, showing the atom-labllling scheme.

**Fig. 2.**
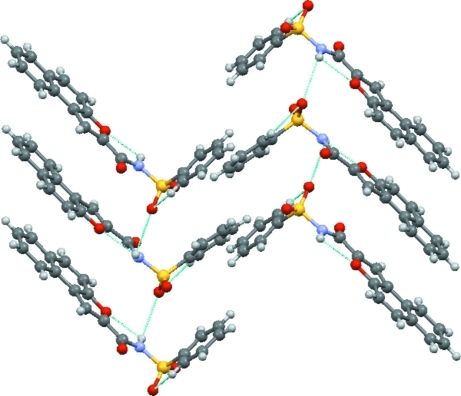
Molecular packing in the title compound. Hydrogen bonds are shown as dashed lines.

## supplementary crystallographic information

### Comment

Aryl Acyl sulfonamides are known as a potent antitumor agent against a broad spectrum of human tumor xenografts in nude mice. Further,the title compound exhibits antibacterial and antifungal activities (our unpublished results). In order to study the effect of the ring substituents on the solid-state structures of *N*-naphthofuroyl-sulfonamides, in the present work the structure of *N*-(Naphthofuroyl)benzenesulfonamide has been determined. The title compound (I) crystallizes in Monoclinic *P*2_1_/*c* space group compared to *N*-(benzoyl)benzenesulfonamide (II) (Gowda *et al.*, 2009) and *N*-(Phenylsulfonyl)acetamide (III)(Gowda *et al.*, 2010) which crystallizes in Triclinic P-1 and Tetragonal P4_3_space groups respectively. In III, the packing of molecules is linked by N—H···.O(C) hydrogen bonds and in II by N—H···.O(S) bonds, whereas in I, the molecules are linked by intermolecular N—H···.O(S) hydrogen bonds. Intramolecular C—H···..O(S) and *N*—*H*···..*O*(furan) interactions are also observed.The molecules are twisted at S atoms with the C—S(O2)—NH—C(O) torsion angle of -65.2 (2)°, compared to the values of -66.9 (3)° in (II) and -58.8 (4)° in (III). The dihedral angle between the benzene ring and the naphthofuran ring in (I) is 83.3 (1)°, compared to 80.3 (1)° observed between the two benzene rings in (II) and 38.7 (0)° in (III) between the benzene ring and the mean plane of CH3 fragement respectively. The packing of the molecules *via* intermolecular N—H···.O(S) hydrogen bonds is shown in Fig. 2.

### Experimental

The title compound was prepared by refluxing a mixture of naphthofuran-2-carboxylic acid (10 mmol),benzenesulfonamide(10 mmol) and phosphorous oxychloride for 1 h on a water bath.The resultant mixture was cooled and poured into ice cold water. The solid, *N*-(Naphthofuroyl)benzenesulfonamide obtained was filtered, washed thoroughly with water and then dissolved in sodium bicarbonate solution. The compound was later reprecipitated by acidifying the filtered solution with dilute hydrochloric acid. The filtered and dried compound was recrystallized to constant melting point. The compound was characterized by its characteristic carbonyl C=O stretching (1698.2 cm-1), N—H stretching (3233.1 cm-1), symmetric SO2(1173.3 cm-1) and asymmetric SO2 (1326.2 cm-1) infrared absorption frequencies. Single crystals suitable for *x*-ray diffraction were grown from a slow evaporation of its ethanolic solution at room temperature.

### Refinement

The H atom of the NH group was located in a difference map and later restrained to N—H = 0.86 (1)%A. The other H atoms were positioned with idealized geometry using a riding model with C—H = 0.93 Å. All H atoms were refined with isotropic displacement parameters (set to 1.2 times of the *U*_eq_ of the parent atom).

### Crystal data

**Table:**

C_19_H_13_NO_4_S	*F*(000) = 728
*M**_r_* = 351.36	1.447 Mg m-3
Monoclinic, *P*2_1_/*c*	*D*_x_ = 1.447 Mg m^−^^3^
Hall symbol: -P 2ybc	Mo *K*α radiation, λ = 0.71073 Å
*a* = 13.8504 (10) Å	Cell parameters from 2986 reflections
*b* = 12.2166 (8) Å	θ = 2.2°
*c* = 9.7164 (6) Å	µ = 0.23 mm^−^^1^
β = 101.248 (2)°	*T* = 299 K
*V* = 1612.48 (19) Å^3^	Prism, colourless
*Z* = 4	0.35 × 0.3 × 0.25 mm

### Data collection

**Table:**

Bruker APEXII CCD area-detector diffractometer	2986 independent reflections
Radiation source: fine-focus sealed tube	2394 reflections with *I* > 2σ(*I*)
graphite	*R*_int_ = 0.027
phi and scans	θ_max_ = 25.5°, θ_min_ = 2.9°
Absorption correction: multi-scan (*SADABS*; Sheldrick, 1996)	*h* = −16→16
*T*_min_ = 0.924, *T*_max_ = 0.945	*k* = −14→14
15289 measured reflections	*l* = −11→11

### Refinement

**Table:**

Refinement on *F*^2^	Secondary atom site location: difference Fourier map
Least-squares matrix: full	Hydrogen site location: inferred from neighbouring sites
*R*[*F*^2^ > 2σ(*F*^2^)] = 0.041	H atoms treated by a mixture of independent and constrained refinement
*wR*(*F*^2^) = 0.133	*w* = 1/[σ^2^(*F*_o_^2^) + (0.0827*P*)^2^ + 1.1854*P*] where *P* = (*F*_o_^2^ + 2*F*_c_^2^)/3
*S* = 0.85	(Δ/σ)_max_ = 0.001
2986 reflections	Δρ_max_ = 0.25 e Å^−^^3^
231 parameters	Δρ_min_ = −0.38 e Å^−^^3^
1 restraint	Extinction correction: *SHELXL*
Primary atom site location: structure-invariant direct methods	Extinction coefficient: 0.0054 (12)

### Special details

**Table:**

Geometry. All e.s.d.'s (except the e.s.d. in the dihedral angle between two l.s. planes) are estimated using the full covariance matrix. The cell e.s.d.'s are taken into account individually in the estimation of e.s.d.'s in distances, angles and torsion angles; correlations between e.s.d.'s in cell parameters are only used when they are defined by crystal symmetry. An approximate (isotropic) treatment of cell e.s.d.'s is used for estimating e.s.d.'s involving l.s. planes.
Refinement. Refinement of *F*^2^ against ALL reflections. The weighted *R*-factor *wR* and goodness of fit *S* are based on *F*^2^, conventional *R*-factors *R* are based on *F*, with *F* set to zero for negative *F*^2^. The threshold expression of *F*^2^ > σ(*F*^2^) is used only for calculating *R*-factors(gt) *etc*. and is not relevant to the choice of reflections for refinement. *R*-factors based on *F*^2^ are statistically about twice as large as those based on *F*, and *R*- factors based on ALL data will be even larger.

### Fractional atomic coordinates and isotropic or equivalent isotropic displacement parameters (Å^2^)

**Table:**

	*x*	*y*	*z*	*U*_iso_*/*U*_eq_	
H1N	0.8221 (19)	0.1816 (19)	0.346 (3)	0.053 (8)*	
S1	0.72588 (4)	0.25754 (5)	0.18450 (6)	0.0566 (2)	
N1	0.82025 (14)	0.24177 (18)	0.3158 (2)	0.0527 (5)	
O1	0.69636 (15)	0.14945 (17)	0.1467 (2)	0.0910 (7)	
O2	0.75502 (14)	0.3299 (2)	0.08507 (18)	0.0853 (7)	
O3	0.85990 (12)	0.42070 (13)	0.35980 (18)	0.0626 (5)	
O4	0.96215 (10)	0.17651 (11)	0.52390 (14)	0.0448 (4)	
C1	1.14610 (14)	0.26862 (18)	0.8155 (2)	0.0430 (5)	
C2	1.18623 (16)	0.3662 (2)	0.8774 (2)	0.0545 (6)	
H2	1.1644	0.4332	0.8376	0.065*	
C3	1.25770 (18)	0.3628 (3)	0.9968 (2)	0.0663 (7)	
H3	1.2841	0.4278	1.0377	0.08*	
C4	1.29114 (18)	0.2631 (3)	1.0575 (3)	0.0703 (8)	
H4	1.3395	0.2621	1.1387	0.084*	
C5	1.25403 (17)	0.1678 (3)	0.9995 (2)	0.0636 (7)	
H5	1.2771	0.102	1.0416	0.076*	
C6	1.18038 (15)	0.16646 (19)	0.8755 (2)	0.0488 (5)	
C7	1.14042 (17)	0.0666 (2)	0.8153 (2)	0.0566 (6)	
H7	1.1644	0.0011	0.8573	0.068*	
C8	1.06823 (17)	0.06330 (19)	0.6984 (2)	0.0550 (6)	
H8	1.0426	−0.0025	0.6593	0.066*	
C9	1.03480 (14)	0.16388 (17)	0.6404 (2)	0.0425 (5)	
C10	1.06976 (14)	0.26419 (16)	0.6922 (2)	0.0395 (4)	
C11	1.01546 (14)	0.34374 (17)	0.6020 (2)	0.0412 (4)	
H11	1.0225	0.4194	0.609	0.049*	
C12	0.95188 (13)	0.28806 (17)	0.5044 (2)	0.0404 (4)	
C13	0.87507 (14)	0.32539 (18)	0.3889 (2)	0.0430 (5)	
C14	0.63289 (15)	0.3220 (2)	0.2518 (2)	0.0502 (5)	
C16	0.4993 (2)	0.3091 (5)	0.3712 (4)	0.1040 (13)	
H16	0.4601	0.2673	0.4186	0.125*	
C15	0.57551 (19)	0.2612 (3)	0.3241 (3)	0.0725 (8)	
H15	0.5888	0.1872	0.3409	0.087*	
C19	0.6169 (2)	0.4313 (3)	0.2335 (4)	0.0876 (9)	
H19	0.6567	0.4741	0.1882	0.105*	
C17	0.4807 (3)	0.4141 (5)	0.3499 (5)	0.1213 (18)	
H17	0.4271	0.445	0.3801	0.146*	
C18	0.5377 (3)	0.4776 (4)	0.2854 (5)	0.1210 (15)	
H18	0.5249	0.5522	0.275	0.145*	

### Atomic displacement parameters (Å^2^)

**Table:**

	*U*^11^	*U*^22^	*U*^33^	*U*^12^	*U*^13^	*U*^23^
S1	0.0409 (3)	0.0788 (5)	0.0460 (3)	0.0105 (3)	−0.0012 (2)	−0.0159 (3)
N1	0.0427 (10)	0.0551 (13)	0.0552 (11)	0.0046 (8)	−0.0028 (8)	−0.0053 (9)
O1	0.0699 (12)	0.0851 (14)	0.1006 (15)	0.0202 (10)	−0.0264 (11)	−0.0464 (12)
O2	0.0582 (10)	0.154 (2)	0.0440 (9)	0.0078 (12)	0.0119 (8)	0.0091 (11)
O3	0.0571 (9)	0.0564 (10)	0.0672 (10)	−0.0011 (8)	−0.0051 (8)	0.0136 (8)
O4	0.0406 (7)	0.0441 (8)	0.0477 (8)	0.0006 (6)	0.0034 (6)	−0.0032 (6)
C1	0.0329 (10)	0.0591 (13)	0.0383 (10)	−0.0002 (8)	0.0104 (8)	0.0039 (9)
C2	0.0465 (12)	0.0679 (15)	0.0478 (12)	−0.0094 (10)	0.0056 (9)	0.0003 (10)
C3	0.0515 (13)	0.092 (2)	0.0534 (13)	−0.0177 (13)	0.0049 (11)	−0.0072 (13)
C4	0.0443 (13)	0.118 (2)	0.0443 (13)	−0.0070 (14)	−0.0007 (10)	0.0093 (14)
C5	0.0421 (12)	0.098 (2)	0.0498 (13)	0.0085 (13)	0.0078 (10)	0.0199 (13)
C6	0.0367 (10)	0.0675 (14)	0.0442 (11)	0.0060 (9)	0.0127 (9)	0.0108 (10)
C7	0.0541 (13)	0.0563 (14)	0.0599 (13)	0.0134 (11)	0.0124 (11)	0.0145 (11)
C8	0.0574 (13)	0.0458 (13)	0.0615 (13)	0.0057 (10)	0.0104 (11)	0.0033 (10)
C9	0.0364 (10)	0.0466 (11)	0.0444 (10)	0.0023 (8)	0.0082 (8)	0.0011 (9)
C10	0.0319 (9)	0.0480 (11)	0.0392 (10)	0.0002 (8)	0.0084 (8)	0.0028 (8)
C11	0.0387 (10)	0.0414 (11)	0.0436 (10)	−0.0016 (8)	0.0082 (8)	0.0009 (8)
C12	0.0350 (9)	0.0430 (11)	0.0441 (10)	0.0011 (8)	0.0096 (8)	0.0012 (8)
C13	0.0360 (10)	0.0519 (13)	0.0413 (10)	0.0003 (9)	0.0084 (8)	0.0004 (9)
C14	0.0348 (10)	0.0639 (14)	0.0491 (11)	0.0011 (9)	0.0009 (9)	−0.0112 (10)
C16	0.0497 (17)	0.190 (4)	0.075 (2)	−0.017 (2)	0.0200 (15)	−0.028 (3)
C15	0.0501 (14)	0.102 (2)	0.0630 (15)	−0.0128 (13)	0.0048 (12)	−0.0044 (14)
C19	0.0656 (17)	0.072 (2)	0.124 (3)	0.0113 (14)	0.0167 (17)	−0.0006 (18)
C17	0.0517 (18)	0.186 (5)	0.124 (3)	0.007 (3)	0.011 (2)	−0.077 (3)
C18	0.091 (3)	0.094 (3)	0.170 (4)	0.035 (2)	0.007 (3)	−0.037 (3)

### Geometric parameters (Å, °)

**Table:**

S1—O1	1.410 (2)	C7—C8	1.360 (3)
S1—O2	1.425 (2)	C7—H7	0.93
S1—N1	1.650 (2)	C8—C9	1.393 (3)
S1—C14	1.742 (2)	C8—H8	0.93
N1—C13	1.383 (3)	C9—C10	1.376 (3)
N1—H1N	0.79 (2)	C10—C11	1.422 (3)
O3—C13	1.207 (2)	C11—C12	1.346 (3)
O4—C9	1.369 (2)	C11—H11	0.93
O4—C12	1.379 (2)	C12—C13	1.461 (3)
C1—C2	1.400 (3)	C14—C19	1.360 (4)
C1—C6	1.419 (3)	C14—C15	1.377 (3)
C1—C10	1.436 (3)	C16—C17	1.316 (6)
C2—C3	1.371 (3)	C16—C15	1.362 (5)
C2—H2	0.93	C16—H16	0.93
C3—C4	1.393 (4)	C15—H15	0.93
C3—H3	0.93	C19—C18	1.412 (5)
C4—C5	1.350 (4)	C19—H19	0.93
C4—H4	0.93	C17—C18	1.346 (6)
C5—C6	1.419 (3)	C17—H17	0.93
C5—H5	0.93	C18—H18	0.93
C6—C7	1.418 (3)		
			
O1—S1—O2	120.71 (14)	O4—C9—C10	110.56 (17)
O1—S1—N1	103.77 (12)	O4—C9—C8	124.53 (19)
O2—S1—N1	108.08 (11)	C10—C9—C8	124.91 (19)
O1—S1—C14	108.85 (12)	C9—C10—C11	106.10 (17)
O2—S1—C14	107.57 (12)	C9—C10—C1	119.19 (18)
N1—S1—C14	107.12 (10)	C11—C10—C1	134.70 (19)
C13—N1—S1	125.68 (18)	C12—C11—C10	106.48 (18)
C13—N1—H1N	121.3 (19)	C12—C11—H11	126.8
S1—N1—H1N	111.1 (19)	C10—C11—H11	126.8
C9—O4—C12	105.34 (15)	C11—C12—O4	111.50 (17)
C2—C1—C6	119.94 (19)	C11—C12—C13	131.45 (19)
C2—C1—C10	123.80 (19)	O4—C12—C13	117.01 (17)
C6—C1—C10	116.25 (19)	O3—C13—N1	122.6 (2)
C3—C2—C1	119.9 (2)	O3—C13—C12	123.26 (19)
C3—C2—H2	120.1	N1—C13—C12	114.10 (19)
C1—C2—H2	120.1	C19—C14—C15	120.1 (2)
C2—C3—C4	120.7 (2)	C19—C14—S1	120.5 (2)
C2—C3—H3	119.7	C15—C14—S1	119.4 (2)
C4—C3—H3	119.7	C17—C16—C15	120.4 (4)
C5—C4—C3	120.6 (2)	C17—C16—H16	119.8
C5—C4—H4	119.7	C15—C16—H16	119.8
C3—C4—H4	119.7	C16—C15—C14	120.1 (4)
C4—C5—C6	121.1 (2)	C16—C15—H15	119.9
C4—C5—H5	119.4	C14—C15—H15	119.9
C6—C5—H5	119.4	C14—C19—C18	117.7 (3)
C1—C6—C5	117.8 (2)	C14—C19—H19	121.2
C1—C6—C7	120.97 (19)	C18—C19—H19	121.2
C5—C6—C7	121.2 (2)	C16—C17—C18	121.4 (4)
C8—C7—C6	122.3 (2)	C16—C17—H17	119.3
C8—C7—H7	118.8	C18—C17—H17	119.3
C6—C7—H7	118.8	C17—C18—C19	120.2 (4)
C7—C8—C9	116.4 (2)	C17—C18—H18	119.9
C7—C8—H8	121.8	C19—C18—H18	119.9
C9—C8—H8	121.8		
			
O1—S1—N1—C13	179.8 (2)	C6—C1—C10—C11	179.5 (2)
O2—S1—N1—C13	50.5 (2)	C9—C10—C11—C12	0.6 (2)
C14—S1—N1—C13	−65.2 (2)	C1—C10—C11—C12	−178.9 (2)
C6—C1—C2—C3	−0.8 (3)	C10—C11—C12—O4	−0.9 (2)
C10—C1—C2—C3	178.7 (2)	C10—C11—C12—C13	177.01 (19)
C1—C2—C3—C4	0.1 (4)	C9—O4—C12—C11	0.8 (2)
C2—C3—C4—C5	0.2 (4)	C9—O4—C12—C13	−177.41 (15)
C3—C4—C5—C6	0.2 (4)	S1—N1—C13—O3	−2.0 (3)
C2—C1—C6—C5	1.1 (3)	S1—N1—C13—C12	177.46 (15)
C10—C1—C6—C5	−178.44 (17)	C11—C12—C13—O3	3.4 (3)
C2—C1—C6—C7	179.6 (2)	O4—C12—C13—O3	−178.81 (18)
C10—C1—C6—C7	0.0 (3)	C11—C12—C13—N1	−176.1 (2)
C4—C5—C6—C1	−0.8 (3)	O4—C12—C13—N1	1.7 (2)
C4—C5—C6—C7	−179.3 (2)	O1—S1—C14—C19	−147.8 (2)
C1—C6—C7—C8	0.1 (3)	O2—S1—C14—C19	−15.4 (2)
C5—C6—C7—C8	178.5 (2)	N1—S1—C14—C19	100.6 (2)
C6—C7—C8—C9	−0.3 (3)	O1—S1—C14—C15	32.2 (2)
C12—O4—C9—C10	−0.4 (2)	O2—S1—C14—C15	164.60 (19)
C12—O4—C9—C8	179.38 (19)	N1—S1—C14—C15	−79.4 (2)
C7—C8—C9—O4	−179.39 (19)	C17—C16—C15—C14	−1.1 (5)
C7—C8—C9—C10	0.4 (3)	C19—C14—C15—C16	3.4 (4)
O4—C9—C10—C11	−0.1 (2)	S1—C14—C15—C16	−176.6 (2)
C8—C9—C10—C11	−179.9 (2)	C15—C14—C19—C18	−2.7 (4)
O4—C9—C10—C1	179.47 (15)	S1—C14—C19—C18	177.3 (3)
C8—C9—C10—C1	−0.3 (3)	C15—C16—C17—C18	−2.0 (6)
C2—C1—C10—C9	−179.43 (19)	C16—C17—C18—C19	2.7 (7)
C6—C1—C10—C9	0.1 (3)	C14—C19—C18—C17	−0.3 (6)
C2—C1—C10—C11	0.0 (3)		

### Hydrogen-bond geometry (Å, °)

**Table:**

*D*—H···*A*	*D*—H	H···*A*	*D*···*A*	*D*—H···*A*
N1—H1N···O4	0.79 (3)	2.33 (3)	2.653 (2)	106 (2)
C19—H19···O2	0.93	2.55	2.890 (4)	102.
N1—H1N···O2^i^	0.79 (3)	2.66 (1)	3.057 (2)	113 (2)

Symmetry codes: (i) *x*, −*y*+1/2, *z*+1/2.
